# RedEfish: Generation of the Polycistronic mScarlet: GSG-T2A: Ttpa Zebrafish Line

**DOI:** 10.3390/antiox10060965

**Published:** 2021-06-16

**Authors:** Brian Head, Jane La Du, Carrie Barton, Jie Zhang, Carmen Wong, Emily Ho, Robyn L. Tanguay, Maret G. Traber

**Affiliations:** 1 Linus Pauling Institute, Oregon State University, Corvallis, OR 97331, USA or brian.head@nih.gov (B.H.); catherin@cau.edu.cn (J.Z.); carmen.wong@oregonstate.edu (C.W.); emily.ho@oregonstate.edu (E.H.); 2 Molecular and Cell Biology Program, Oregon State University, Corvallis, OR 97331, USA; 3 Department of Environmental Toxicology, College of Agricultural Sciences, Oregon State University, Corvallis, OR 97331, USA; Jane.LaDu@oregonstate.edu (J.L.D.); Carrie.Barton@oregonstate.edu (C.B.); robyn.tanguay@oregonstate.edu (R.L.T.); 4 College of Science, China Agriculture University, Beijing 100083, China; 5 School of Biological and Population Health Sciences, College of Public Health, Oregon State University, Corvallis, OR 97331, USA

**Keywords:** vitamin E, alpha-tocopherol, alpha-tocopherol transfer protein (TTPA)

## Abstract

The vitamin E regulatory protein, the alpha-tocopherol transfer protein (Ttpa), is necessary for zebrafish embryo development. To evaluate zebrafish embryo Ttpa function, we generated a fluorescent-tagged zebrafish transgenic line using CRISPR-Cas9 technology. One-cell stage embryos (from Casper (colorless) zebrafish adults) were injected the mScarlet coding sequence in combination with cas9 protein complexed to single guide RNA molecule targeting 5′ of the ttpa genomic region. Embryos were genotyped for proper insertion of the mScarlet coding sequence, raised to adulthood and successively in-crossed to produce the homozygote RedEfish (mScarlet: GSG-T2A: Ttpa). RedEfish were characterized by in vivo fluorescence detection at 1, 7 and 14 days post-fertilization (dpf). Fluorescent color was detectable in RedEfish embryos at 1 dpf; it was distributed throughout the developing brain, posterior tailbud and yolk sac. At 7 dpf, the RedEfish was identifiable by fluorescence in olfactory pits, gill arches, pectoral fins, posterior tail region and residual yolk sac. Subsequently (14 dpf), the mScarlet protein was found in olfactory pits, distributed throughout the digestive tract, along the lateral line and especially in caudal vertebrae. No adverse morphological outcomes or developmental delays were observed. The RedEfish will be a powerful model to study Ttpa function during embryo development.

## 1. Introduction

Vitamin E (VitE, α-tocopherol) is necessary for embryonic development [1]. Low maternal VitE in human serum and cord blood is linked to prematurity, low birth weight [2], increased risk of miscarriage [3] and intrauterine growth restriction [4]. Preterm babies were found with lower VitE relative to term babies of mothers with similar cord blood VitE levels [5] and circulating VitE in women at early and late term was positively correlated to fetal growth and decreased risk for low birth weight [6].

VitE deficiency in humans can be caused by genetic defects in the α-tocopherol transfer protein (TTPA (Ttpa in zebrafish), note that human, mouse and rat proteins are indicated by all capital letters, while zebrafish proteins are indicated by a capitalized first letter) [7,8]. TTPA is localized primarily in hepatocytes, where it facilitates VitE trafficking from recycling endosomes to the membrane in exchange for inositol-triphosphate (Ins3P) [9], the transfer of VitE to nascent lipoproteins is then facilitated by ABCA1 [10], an ATP-dependent transporter. In addition to the liver, TTPA is also expressed in the human yolk sac [11] and in placental uterine lining at the site of implantation [12]. Limited information exists, however, about the role of Ttpa in the developing embryo.

Zebrafish embryos are a frequently used model for developmental biology because they develop externally are transparent, easy to maintain and are produced in large quantities [13]. The zebrafish embryo has been used as a powerful tool to study numerous human-health related conditions, including birth defects [14,15,16,17,18,19]. Importantly, early development gene expression networks are highly conserved between zebrafish and humans [20], especially the sequence for *ttpa* (64.3% protein sequence homology between zebrafish and human). Zebrafish require both vitamins E and C, so are highly relevant for antioxidant research [21,22]. Thus, the VitE-deficient zebrafish embryo model (E– embryos) allows us to evaluate developmental dysregulation in a highly relevant model. Our research using E– embryos has demonstrated that VitE prevents lipid peroxidative damage [23] and the ensuing metabolic responses aimed to repair the damage [24]. We found that these metabolic responses are detrimental because they deplete critical nutrients and waste limited energy resources needed for embryo development [24,25,26]. Indeed, supplementation of the E– embryos with VitE, or glucose, rescued the animals [24,25]. These metabolic abnormalities were associated with dysregulation of gene networks under the control of mechanistic target of rapamycin (mTORC1) [27]. Further, E– embryos show significant morphologic abnormalities as early as 12 hpf [28]. VitE was necessary for brain (fore, mid and hindbrain) development at 12 hpf and later (24 hpf) for the dorsal root ganglia and notochord formations [28]. The *ttpa* gene is expressed in the zebrafish brain ventricle borders, eye and tailbud at 24 h post-fertilization (hpf) [28,29]. Translation- and splice-blocking morpholinos preventing Ttpa synthesis caused 100% lethality in zebrafish embryos by 24 hpf [29]. These findings suggest that TTPA and VitE have important functions in the embryo separate from placental implantation. During embryogenesis, TTPA could potentially function to (1) transfer VitE from the yolk to the developing embryo, (2) facilitate VitE transport from the placenta to the embryo and (3) regulate VitE trafficking in cells of the developing nervous system in the embryo.

We hypothesize that studies of Ttpa localization in the embryo will provide critical information concerning its essential function to deliver VitE to target sites. To test this hypothesis, we developed a transgenic zebrafish model containing a red fluorophore tagged Ttpa. CRISPR-cas9 technology was used to knock-in a genomic mScarlet sequence 5′ upstream of the *ttpa* genomic sequence separated by a self-cleaving viral peptide sequence (GSG-T2A). We used mScarlet because it is a powerful red fluorophore with more intensity (0.54 quantum yield units) and longer lifetime (3.1 ns) than other known red fluorescent proteins [30]. The mScarlet protein is not cytotoxic and has been shown previously to be effective for creation of transgenic zebrafish lines [31]. We report herein the creation of the RedEfish (mScarlet: GSG-T2A: Ttpa) transgenic line.

## 2. Materials and Methods

### 2.1. Zebrafish Husbandry and Strains

All experimental protocols and methods were carried out in accordance with the animal use and care protocol (# 5068) approved by the Institutional Animal Care and Use Committee at Oregon State University. 5D tropical strain and Tg zebrafish (Casper; *mitfa*^w2/w2^, *mpv17*^a9/a9^) strain (hereafter called Casper) [32] and c269 (Tg(UAS-E1b:NfsBmCherry)^c264/+^) strain (hereafter called c269) [33] were reared in Sinnhuber Aquatic Research Laboratory at Oregon State University under standard laboratory conditions of 28 °C on a 14 h light/10 h dark photoperiod according to standard zebrafish breeding protocols [34]. The 5D tropical strain and the c269Tg reporter line zebrafish were used as additional negative and positive controls for experiments, noted throughout.

### 2.2. Construction of Donor Plasmid and Guide RNA Vectors for Knock-In

**Figure 1:**
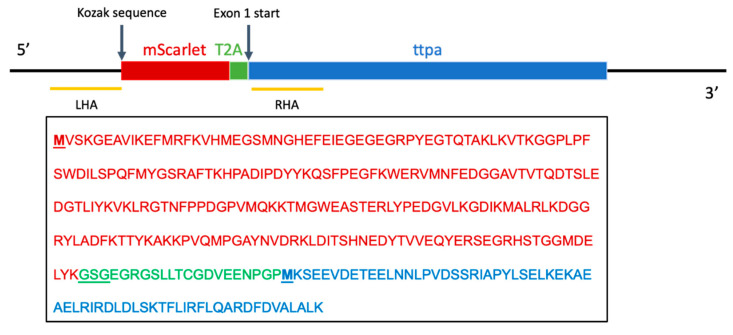
Knock-in strategy and designed protein sequence. LHA refers to the left homology arm and RHA refers to the right homology arm of the donor plasmid containing the mScarlet coding sequence. Amino acid sequences are denoted by color to indicate protein: mScarlet (red), GSG-T2A linker site (green) and exon 1 of *ttpa* (blue).

The donor plasmid containing homology arms to the *ttpa* genomic sequence (GenBank:BX248497, RefSeq:NM_199731), mScarlet sequence, Gly-Ser-Gly linker (GSG) sequence and 2A self-cleaving peptide (T2A) was constructed and supplied by In Vivo Biosystems, Inc. (Eugene, OR, USA). Constructs were assembled by Gibson ligation and confirmed by digest at restriction sites Pvul and Pvull, followed by sequencing. A single guide RNA (sgRNA) sequence was designed with the custom designed short CRISPR RNA (crRNA) sequence fused to the scaffold trans-activating CRISPR RNA (tracRNA) sequence (Figure 1).

### 2.3. Microinjection

sgRNA and cas9 protein were complexed and coinjected into one-cell stage zebrafish embryos. Approximately 1 nL of injection mix (10.5 μM sgRNA, 500 ng/μL cas9, 50 ng/μL donor plasmid and 50 μM NU7441 in 1% DMSO) was injected directly into each embryo. Embryos were then placed on ice-cold 1.5% agarose for no more than 15 min. NU7441 and cold exposure after injection were used to improve knock-in efficiency of mScarlet sequence by homology-directed repair mechanisms (HDR) [35]. NU7441 in 1% DMSO (50 μM) injected at the levels described did not have deleterious effects on normal zebrafish embryos, as assessed by morbidity, mortality and behavioral outcomes at 5 days post-fertilization (dpf) [36]. Injected embryos were collected, staged, and incubated in standard embryo media ((EM), 15 mM NaCl, 0.5 mM KCl, 1 mM MgSO_4_, 0.15 mM KH_2_PO_4_, 0.05 mM Na_2_HPO_4_, 1 mM CaCl_2_ and NaHCO_3_ in fish system water).

### 2.4. Mutation Detection and Insertion Mapping

Genomic DNA (gDNA) was extracted from injected embryos (*n* = 32) at 5 dpf after each trial, as described [37]. Embryos were euthanized with tricaine (MS222, ethyl 3-aminobenzoate methanesulfonate salt (Sigma Aldrich, St. Louis, MO, USA)) in accordance with animal care and use guidelines, placed in 50 μL 50 mM NaOH and incubated at 95 °C for 15 min. DNA extraction was stopped with 10 μL 1M TRIS pH 8.0, and stored in 4 °C until use.

PCR reactions were performed with primers designed to capture the complete insertion region (Table 1). Target regions were amplified by PCR and sequenced by Sanger Sequencing at the Center for Genome Research and Biocomputing (CGRB) at Oregon State University. Adult zebrafish were fin clipped at 3 months, gDNA extracted and PCR performed as described above. Identified insertion carriers were out-crossed with Casper zebrafish to produce F1 heterozygotes Tg(mScarlet:ttpa; *mScarlet*^+/−^, *mitfa*^w2/w2^, *mpv17*^a9/a9^), which were then successively in-crossed to produce F2 heterozygotes and F3 homozygotes (hereafter called RedEfish).

### 2.5. In Vivo Fluorescence Detection

Live embryo imaging was conducted using a Keyence BZ-x700 All-in-one microscope with a 2× or 10× objective and 644 nm filter. Live embryos were mounted in 3% methylcellulose at room temperature in 35 mm glass bottom MatTek dishes and covered in embryo media and tricaine to anesthetize live embryos. Image adjustments, including cropping, brightness and contrast were performed uniformly using Adobe Photoshop.

### 2.6. Egg Quality and Embryo Morphological Assessment

RedEfish embryos were collected, staged, and incubated in standard EM. Egg traits were assessed by egg yolk diameter followed by egg volume estimated based on the volume of an ellipsoid using the equation [38,39]:

$$\mathrm{Volume}=\left(4/3\right)\pi \;\times \left(\mathrm{small}\;\mathrm{axis}\;\mathrm{radius}\right)\times {\left(\mathrm{large}\;\mathrm{axis}\;\mathrm{radius}\right)}^{2}$$

At 24 hpf and 5 dpf, RedEfish and Casper embryos were assessed for morbidity and mortality outcomes, as described [36,40]. RedEfish and Casper 5 dpf larval α- and γ-tocopherol concentrations were determined using high-performance liquid chromatography with electrochemical detection, as described with comparison to authentic standards [41].

### 2.7. Immunolocalization and Western Blot Analysis

Distribution of mScarlet protein was assessed in embryos (3 dpf) using the polyclonal antibody to red fluorescent protein (anti-RFP) (anti-rabbit RFP, 1:3000; abcam, ab62341 (Cambridge, MA, USA)). Monoclonal antibodies generated against acetylated tubulin (mouse anti-goat, 1:4000; Sigma Aldrich (St. Louis, MO, USA)) were used to label axons in the developing embryo as a positive control. Embryos (*n* = 15 per group) at the described time points were fixed overnight in 4% paraformaldehyde (PFA) in phosphate-buffered saline (PBS), washed in PBS the following morning and stored at 4 °C in PBS and 0.1% sodium azide until use. Fixed embryos were washed in PBS + 0.1% Tween20 (PBST), permeabilized with 0.005% trypsin in PBS on ice for 5 min, rinsed with PBST and post-fixed in 4% PFA. Permeabilized embryos were blocked in 10% normal goat serum in PBS + 0.5% Triton X-100 for an hour followed by incubation with the primary antibody overnight at 4 °C in 1% normal goat serum in PBS and 0.5% Triton X-100. Embryos were washed 4 times for 30 min in PBST, incubated with secondary antibody (Alexa-488 goat anti-mouse, 1:1000; ThermoFisher (Carlsbad, CA, USA)) for 2 h and washed 4 times for 30 min in PBST.

Distribution of Ttpa protein was assessed in zebrafish embryos (5 dpf) using the polyclonal anti-TTPA antibody (rabbit anti-zebrafish Ttpa (L6308, Antibodies Inc., Davis, CA, USA), 1:300). Embryos previously fixed in 4% PFA were washed in PBST and stored in 100% methanol. Samples were rehydrated and treated with 10 mg/mL proteinase K in PBST. Samples were then blocked in 5% normal goat serum and 3 mg/mL bovine serum albumin in PBST followed by incubation with the primary antibody. Embryos were washed, incubated with secondary antibody (Alexa-594 goat anti-rabbit, 1:1000; ThermoFisher (Carlsbad, CA, USA)), washed and visualized, as described [42].

All fixed embryo imaging was conducted using Keyence BZ-x700 All-in-one microscope with a 2×, 10× or 20× objective and 470 nm or 694 nm filter, as described above. Image adjustments, including cropping, brightness and contrast were performed uniformly using Adobe Photoshop.

For Western blots, protein was extracted from pooled (*n* = 25) embryos (24 hpf) in RIPA buffer supplemented with protease and protein phosphatase inhibitors (Calbiochem, La Jolla, CA, USA). Rat liver extract and extracts of c269 embryos were used as positive controls for Ttpa and RFP. Extracted protein (20 μg) was subjected to SDS-Page and processed by immunoblotting. Antibodies used for Western blotting were anti-ATP synthase F1 subunit α (1:1000, ab110273), anti-Ttpa (1:250, L6308), anti-RFP (1:1000, ab62341), goat anti-rabbit IgG-HRP and goat anti-mouse IgG-HRP secondary antibodies (1:10,000). Nonspecific protein binding was blocked using Tris-buffered saline with 0.1% Tween 20 (TBST) containing 5% nonfat dry milk. All antibodies were diluted in TBST containing 1% bovine serum albumin. Proteins were visualized by SuperSignal^TM^ West Femto Chemiluminescent Substrate (ThermoFisher, Carlsbad, CA, USA) and quantified using the Bio-Rad Image System (Hercules, CA, USA).

### 2.8. Statistical Methods

Statistical differences between groups were assessed using Student’s *t*-test (Prism 6.0, GraphPad, La Jolla, CA, USA). Statistical significance between differences was set at *p* < 0.05. Differences between reproductive output and egg yolk volume in 3 hpf embryos was assessed with *t*-tests between Casper and RedEfish. Similarly, the vitamin E contents of the embryos are reported as comparisons between the two fish lines. Additionally, tocopherol data were logarithmically transformed data for each fish line, then 2-factor ANOVA was followed by Holm–Sidak’s multiple comparisons test to allow for comparison between α- and γ-tocopherol concentrations in each fish line. Data are reported as means ± standard deviation.

## 3. Results

Mosaicism in the founder generation was not immediately apparent by fluorescent imaging. PCR genotyping was utilized to identify sequence expressing fish. About 7.5% (*n* = 19 of 254) of 3 months old (mo) adults screened were identified as transgenic; one screened adult (male) retained the mScarlet:T2A construct. Success was defined as integration of the mScarlet sequence 5′ of *ttpa* without erroneous insertions or deletions (INDELs), as determined by Sanger Sequencing. The mosaic founder male was outcrossed with female Casper zebrafish (*mitfa*^w2/w2^, *mpv17*^a9/a9^) to produce F1 embryos. F1 embryos were raised to 3 months for further in-crossing with the F0 mosaic founder. This in-cross produced F2 heterozygotes, as identified by PCR and sequencing. In vivo fluorescence of heterozygotes was apparent by 5 dpf, however, the yolk sac remnants were a significant source of autofluorescence. F3 in-crosses were performed to generate homozygotes. Sequencing of heterozygote embryos indicated two gene products, one containing the mScarlet:*ttpa* construct and the other containing the unmodified *ttpa* exon 1 (Supplementary Figure S1). Sequencing about the targeted region show no INDELs either in the 5′ region of mScarlet or in the 5′ region of *ttpa* exon1 (Supplementary Figure S1).

**Figure 2:**
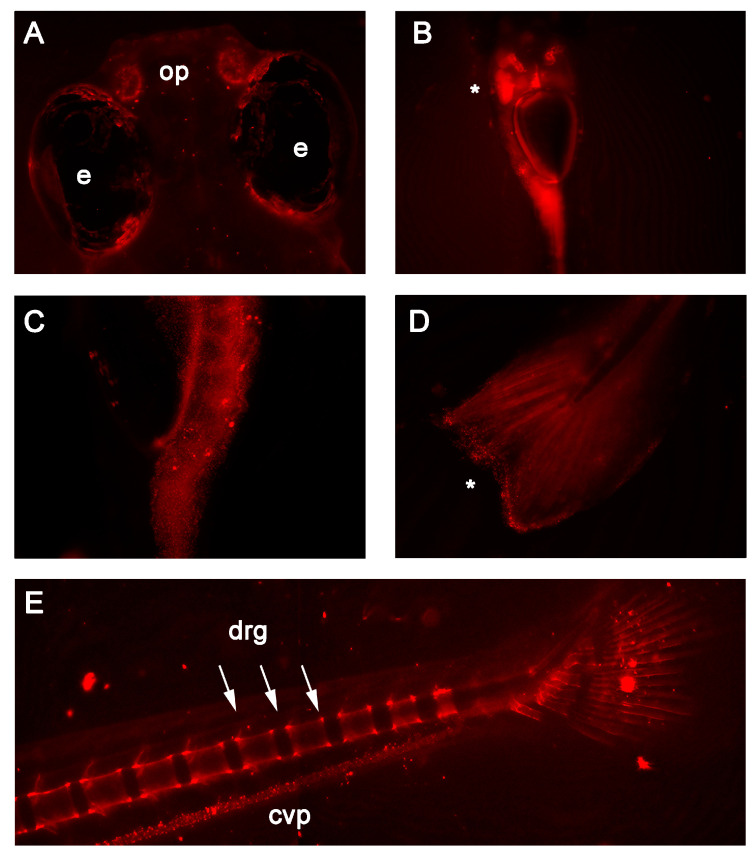
In vivo fluorescence detected in 7 and 14 days post-fertilization RedEfish. (**A**) mScarlet expression was unique to the olfactory pits (op) found in the RedEfish; however, some autofluorescence was detected in the pigment cells surrounding the eyes (e) in all embryos. (**B**) mScarlet expression was identified in the digestive tract, specifically the liver (as indicated by *). (**C**) More punctate expression was observed in the anterior gut region (ventral embryo positioning). (**D**) Punctate expression was observed also in the tail fin (as indicated by *). **(E**) By 14 dpf, mScarlet expression was identified in the caudal vertebrae and dorsal root ganglia (drg) and was detected in the caudal vein plexus (cpv). Some autofluorescence was noted in both RedEfish and Casper larvae.

Fluorescent color was detectable in RedEfish embryos at 1 dpf; it was distributed throughout the developing brain, posterior tailbud and largely in the yolk sac (data not shown). In vivo fluorescence was more readily detected during the early larval stage (7 dpf). RedEfish express mScarlet in the olfactory pits (Figure 2A), throughout the liver and digestive tract (Figure 2B,C), and express punctate signals throughout the tail fin and fin edges. By 14 dpf, RedEfish express mScarlet throughout the digestive tract. Most striking, mScarlet expression was found in the caudal vein plexus, caudal vertebrae, and tail fin. The red fluorescence in the vertebrae is localized to dorsal root ganglia (Figure 2E).

Whole mount immunolocalization was performed to validate the presence of mScarlet and TTPA. GSG-T2A linker sites are not 100% efficient peptide cleavage sites, which can cause either (1) mScarlet fused to exon 1 of Ttpa, which may potentially produce fluorescent but nonfunctional protein, or (2) skipped and decrease the production of Ttpa. This phenomenon has been described [43]. RedEfish embryos were fixed in 4% PFA and subjected to antibody staining with an anti-RFP antibody. Casper embryos were used as a negative control, while the c269 allelic variant embryo was used as a positive control; antiacetylated tubulin is used as a control antibody. All embryos are 3 dpf. Rfp was expressed in RedEfish brain, trunk, yolk syncytium and pectoral fins. Rfp was not detected in Casper embryos, but was detected in the brain of positive control c269 embryos (Supplementary Figure S2).

**Figure 3:**
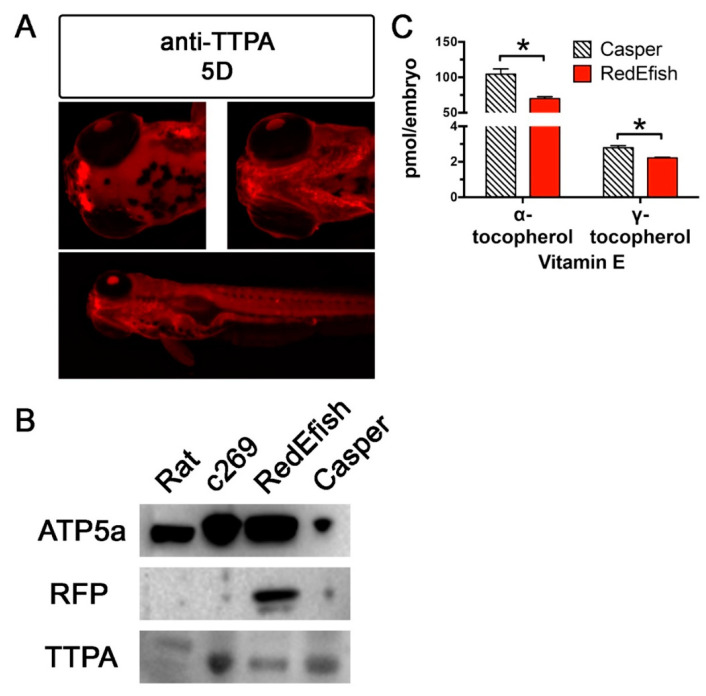
Immunolocalization and Western blot of Ttpa protein in rat liver and zebrafish embryos (c269, RedEfsh and Casper). (**A**) The 5D strain fish expressed Ttpa in olfactory pits, gill arches and gut lining at 5 dpf. Anti-Ttpa was used to detect and validate Ttpa protein expression in 5D embryos initially to determine regionalization followed by in vivo observation. Figure A panels were generated with the BZ-x700 microscope, processed with BZ-X Analyzer Software with image adjustments equally applied across all images in Adobe Photoshop. All images are taken at 2× magnification at the same exposure protocol. This figure was created with Adobe Photoshop, v21.2.1. (**B**) The same anti-Ttp antibody, used in a Western blot, confirmed that Ttpa expression is not affected by the insertion of the mScarlet coding sequence in RedEfish relative to Casper or the c269 transgenic lines. This antibody reacts poorly with rat liver protein. RFP was also detected in the RedEfish embryos, although not detected by blot in the rat liver, c269 or Casper zebrafish embryos. (**C**) Shown are α- and γ-tocopherol concentrations (mean ± standard deviation, pmol/embryo, * = Student’s *t*-test, *p* = 0.002 and *p* = 0.001, respectively) in 5 day old Casper (hatched bars) and RedEfish (red solid bars) embryos (*n* = 5 homogenate pools per group, *n* = 15 embryos per pool). Both RedEfish and Casper embryos contained lower mean γ- as compared with α-tocopherol concentrations; the comparison of logarithmically transformed data for each fish line was significantly different (*p* < 0.0001, Holm-Sidak’s multiple comparisons test).

Immunolocalization with anti-TTPA validated that Ttpa is expressed in the zebrafish embryos (5 dpf) in olfactory pits, gill arches and digestive tract lining (Figure 3A). Western blot analysis was also used to evaluate the success of protein expression in RedEfish. As expected, Ttpa was detected at approximately 32 KDa in RedEfish, Casper and the c269 lines (24 hpf) (Figure 3B). If the mScarlet-Ttpa construct was produced but not hydrolyzed, we anticipated a protein band at ~60 kDa (sum of mScarlet (26.4 kDa) and Ttpa (32.7 kDa)). No band for a larger protein at approximately 60 kDa was observed. An antibody to Rfp was also used to determine if mScarlet protein is expressed in the RedEfish line, although this antibody has not previously been used with mScarlet. The anti-RFP antibody used for immunolocalization detected RFP in the RedEfish embryos, however, did not detect RFP in the c269 embryos.

### Embryo Morphology and Egg Quality Assessment

Egg quality was assessed in F3 RedEfish and Casper embryos at 6 hpf (gastrulation), when *ttpa* is first expressed in zebrafish [28,29]. Egg volume was not significantly different between RedEfish (0.220 ± 0.024 mm^3^) and Casper (0.205 ± 0.023 mm^3^) embryos (*p* = 0.18, *n* = 10 per group). Morbidity and mortality outcomes at 5 dpf were also similar with fewer than 5% of a given clutch experiencing a developmental deformity (*n* = 100 embryos each in 3 independent spawning events). Finally, because of potential modification to Ttpa by insertion of the mScarlet coding sequence, VitE status was assessed in RedEfish and Casper embryos at 5 dpf. Both α- and γ-tocopherol concentrations were significantly lower in RedEfish relative to Casper strain embryos (Figure 3C). Importantly, the RedEfish had lower mean γ- as compared with α-tocopherol concentrations (*p* < 0.0001, Holm–Sidak’s multiple comparisons test), emphasizing that the Ttpa discriminatory capability in the RedEfish remained functional.

## 4. Discussion

We present the RedEfish, a new model for studying Ttpa localization and trafficking. This zebrafish embryo model will be useful to study vertebrate development and as the zebrafish grows to identify the specific tissue and cellular expression of Ttpa. Based on the concept that mScarlet accumulates in cells that synthesize Ttpa, we provide further support that Ttpa is associated with numerous developing embryonic structures, especially throughout the nervous system, circulatory system and digestive tract based on Ttpa presence established via expression of mScarlet. These data also suggest that VitE is required during development by these specific cells in these systems because in humans [44,45] and mice [46,47,48] TTPA’s known function is to traffic α-tocopherol. Measurements of α- and γ-tocopherol concentrations in the RedEfish documented that these fish discriminate between these tocopherols (Figure 3C), as expected for functional Ttpa [49].

Lecithotrophic animals, those that rely on maternal deposited yolk, are an important model for lipid and lipid-soluble vitamin metabolism during yolk utilization. The zebrafish embryo develops separately from its yolk [50,51], which is bound by the yolk syncytial layer (YSL) that secretes nutrients from the yolk into the developing organism. The YSL expresses many of the genes needed for lipid and lipid-soluble vitamin transport [52,53,54] likely facilitating the transfer of VitE from the yolk to the embryo. Zebrafish express the microsomal triglyceride transfer protein (Mttp) immediately after the gastrulation in the YSL, which facilitates apolipoprotein B (apoB) secretion to increase neutral lipid secretion and accumulation throughout the embryo until the yolk depletes [55,56]. Mice similarly require *mttp* to export triglyceride in apoB-containing lipoproteins and *mttp*-null mice die midgestation [57,58]. Defects in *mttp* causing abetalipoproteinemia in humans also cause VitE deficiency [59]. Thus, VitE transport from the yolk likely depends on the lipid metabolic machinery of the YSL. The *ttpa* mRNA was found in zebrafish embryo YSL and early neural structures including the eye, brain and posterior tailbud [28,29]. We report mScarlet fluorescence is detected in the YSL in the zebrafish embryo throughout yolk depletion and is found as punctate expression throughout the larval digestive tract until at least 14 dpf. Ttpa may be involved in retrieving VitE from yolk stores and from the enterocytes upon first feeding at 5 dpf, although this suggestion has not been observed in other embryonic models. We previously reported that VitE-deficiency adversely impacts neural crest cells expressing *sox10* [28], which are known precursors of the enteric nervous system. These data suggest that VitE may be important during enteric neuron maturation.

Ttpa, a cytosolic protein, localizes to recycling endosome, which is also marked by expression of Niemann-Pick C1 and C2 (Npc1 and 2). Both Npc1 and Npc2 are expressed specifically in the zebrafish embryo YSL [60], a metabolically active organ that exists up to 9 dpf [61]. Npc1^-/-^ zebrafish mutants have increased unesterified cholesterol in trunk neuromasts indicating localization of impaired recycling endosomal metabolism and possible colocalization of Ttpa [62], supported by early embryonic mScarlet expression in our line. Indeed, NPC1^-/-^ mice experience negatively impacted hepatic and cortical VitE levels [63], with accumulation of VitE in lysosomal compartments [64]. Additionally, NPC1-like 1 mediates tocopherol uptake by human [65] and mouse [66] intestinal epithelial cells. Our model supports previous findings that Ttpa and Npc likely localize to the same cell or tissue types involving endosomal recycling and cholesterol trafficking, including neuromasts, the lateral line and the intestinal tract especially in 7 dpf embryos.

The mScarlet fluorescence signal was not immediately apparent in the mosaic founder zebrafish nor in the early embryonic stages up to 5 dpf in the heterozygote or homozygote RedEfish. Although some fluorescence was detected during this developmental window, successful transgenesis required molecular validation because of the yolk autofluorescence [67]. The bright yolk sac may be indicative of Ttpa and simultaneous mScarlet expression, however, that outcome is currently unclear. We hypothesized that the presence of VitE separate from yolk-derived transport may stimulate embryonic tissue expression of Ttpa and mScarlet. VitE supplementation increases Ttpa mRNA expression in isolated rat liver after food deprivation [68]. In addition, VitE supplementation stabilizes and prevents degradation of TTPA in HepG2 cells [69]. Punctate protein expression may also be achieved by VitE supplementation because TTPA colocalizes to lysosomes, as indicated in isolated mouse hepatocytes by LAMP1 and fluorescent tocopherol upon supplementation [70]. Feeding 5 dpf embryos induced red fluorescence detected at 7- and 14-dpf, the signal was, however, primarily localized to the gut and digestive tract lining rather than the liver, as expected. Larval zebrafish “guts” remain highly autofluorescent [71], possibly as a result of their microbiome [72]. Further investigation of the RedEfish model should exclude examination of the auto-fluorescent yolk and digestive tract, although these structures are still critical for dissecting the functions of VitE and Ttpa in embryonic development.

Importantly, VitE is associated with nervous system development. Chronically VitE deficient mice [73] and zebrafish [74] experience cognitive dysfunction resulting from brain specific lipid peroxidation. In addition, blocking translation of Ttpa prevented formation of the eye and brain of 24 hpf zebrafish embryos [29]. Ttpa is found in the cerebellum of adult rats [75] and in mice with VitE deficiency caused by deletion of Ttpa exhibit lipofuscin accumulation as a result of oxidative damage specifically in the dorsal root ganglia (DRG) [76]. DRG from Ttpa^-/-^ mice have increased apoptosis [77], causing reduced mechanosensitivity and excitability prevented only by very high levels of VitE supplementation [78]. Additionally, we found that the DRG was disrupted in VitE deficient zebrafish embryos [28]. Knowing this information, we anticipated mScarlet expression as a proxy for Ttpa expression in the DRG of adult zebrafish (Figure 1E). Thus, the mScarlet expression in the DRG of the RedEfish was expected. Nonetheless, we cannot rule out autofluorescence; however, it is unclear what would produce this punctate effect in 21 dpf zebrafish embryos.

mScarlet can be visualized using a fluorescence microscope with an absorbance and emission spectra of 569 nm and 594 nm, respectively. Guide RNA (gRNA) sequences were selected for a region 5′ of the first *ttpa* exon to create a polycistronic 2A construct encoding mScarlet and *ttpa* in tandem. The T2A self-cleaving peptide has the highest cleavage efficiency of the 2A peptides and has been used to produce independent fluorophore and target proteins in immortalized cell lines, and zebrafish embryos [43]. We were able to confirm, using anti-TTPA that Ttpa is expressed in the RedEfish at 24 hpf, and in other transgenic fish lines (c269 and Casper, Figure 3B). We also confirm Rfp by Western blot analysis in the RedEfish (Figure 3B) and by whole mount immunolocalization with the anti-RFP; note that the c269 expresses RFP specifically in the brain [33] (Supplementary Figure S2). With regards to the construct used, the T2A site was chosen due to high “cleavage” efficiency for a 2A self-cleaving peptide sequence in zebrafish [43]. However, 2A sites present a technical challenge as some bi-cistronic proteins remain uncleaved because of ribosomal read-through or even falling-off [79]. Since VitE levels were found to be somewhat lower in RedEfish relative to the Casper line, it appears that some ribosomal skipping may be occurring to produce reduced Ttpa levels and thus reduced VitE levels in the embryo. Western blot analysis did not indicate reduced Ttpa expression nor did it show a larger unhydrolyzed protein consistent with an unhydrolyzed mScarlet and Ttpa. An additional potential mechanism for the observed lower embryonic VitE, is less VitE added to the developing egg by the adult female fish, as has been shown for chickens [80].

In summary, the RedEfish is a new model to study VitE trafficking in early embryonic development in a model organism suitable for further genetic modification and easy observation by external development. To confirm that the reporter construct was effectively integrated and coexpressed with *ttpa*, sequencing of the genomic region, Western blot analysis and immunolocalization was performed. Confirmation that the reporter construct does not impair Ttpa function was also performed by morphologic assessment of 6 hpf embryos and VitE status of 5 dpf embryos. Thus, we provide ample supporting evidence of the proper germ-line transmission of the mScarlet coding sequence 5′ of the first exon of *ttpa*. We report herein a fluorescent tagged Ttpa model to track VitE-dependent and Ttpa-dependent cells at the onset of development in the zebrafish embryo. This model will provide a powerful tool to evaluate the effect of VitE status, as we have previously shown [24,25,26,27,28,81,82] with in vivo detection and fluorescent presentation of the VitE deficient phenotype.

## Figures and Tables

**Table 1:** Primers designed for insertion mapping.

Primer Purpose	Sequence (5′-3′)	Product Size (bp)
Left homology arm F	AGGCGTGTGTCTCTGTAAGG	418
Left homology arm R	GCCGTACATGAACTGAGGGG	
Right homology arm F	CGCTACCTGGCGGACTTC	309
Right homology arm R	AATCCGGGAGGAATCAACAGG	
mScarlet cassette F	CGCTCTGAGAACAACATGACAC	121 no insertion 879 insertion
mScarlet cassette R	GACAAATATGGTGCAATCCGGG	

Primers designed for left and right homology arms were first utilized to identify germ-line transmission of the mScarlet sequence. Successful insertion and validation of mScarlet expression determined with mScarlet cassette primers. All primers designed with NCBI primer Blast. F = forward primer, R = reverse primer.

## Data Availability

Data is contained within the article and supplementary material.

## Supplementary Materials

The following are available online at https://www.mdpi.com/article/10.3390/antiox10060965/s1, Figure S1: Genotyping confirmation indicates successful genomic integration in heterozygote RedEfish, Figure S2: RFP expression confirmation in 3 days post-fertilization (dpf) c269, Casper and RedEfish.
