# Supplementary material for: RedEfish: Generation of the Polycistronic mScarlet: GSG-T2A: Ttpa Zebrafish Line
Source: Antioxidants (Basel). 2021 Jun 16;10(6):965. doi: 10.3390/antiox10060965 (PMC8235169; doi:10.3390/antiox10060965)
Supplement: Supplementary file 1 [file antioxidants-10-00965-s001.zip › antioxidants-1246105-supplementary.pdf]

RedEfish: generation of the polycistronic mScarlet:GSG-T2A:Ttpa zebrafish line

Brian Head [1,2,3], Jane La Du [4], Carrie Barton [4], Jie Zhang [1,5], Carmen Wong [1], Emily Ho [1,6], Robyn L. Tanguay [4]
and Maret G. Traber [1,6,*]

[1] Linus Pauling Institute, Oregon State University, Corvallis, OR 97331, USA; Brian.Head@oregonstate.edu (B.H.); catherin@cau.edu.cn (J.Z.); carmen.wong@oregonstate.edu (C.W.); emily.ho@oregonstate.edu (E.H.)
[2] Molecular and Cell Biology Program, Oregon State University, Corvallis, OR 97331, USA;
[3] current address: National Institutes of Health, National Institute of Child Health and Human Development,
Bethesda, MD 20817, USA; brian.head@nih.gov
[4] Department of Environmental Toxicology, College of Agricultural Sciences, Oregon State University,
Corvallis, OR 97331, USA; Jane.LaDu@oregonstate.edu (J.L.D.); Carrie.Barton@oregonstate.edu (C.B.); robyn.tanguay@oregonstate.edu (R.L.T.)
[5] College of Science, China Agriculture University, Beijing 100083, China
[6] School of Biological and Population Health Sciences, College of Public Health, Oregon State University, Corvallis, OR 97331, USA
* Correspondence: maret.traber@oregonstate.edu

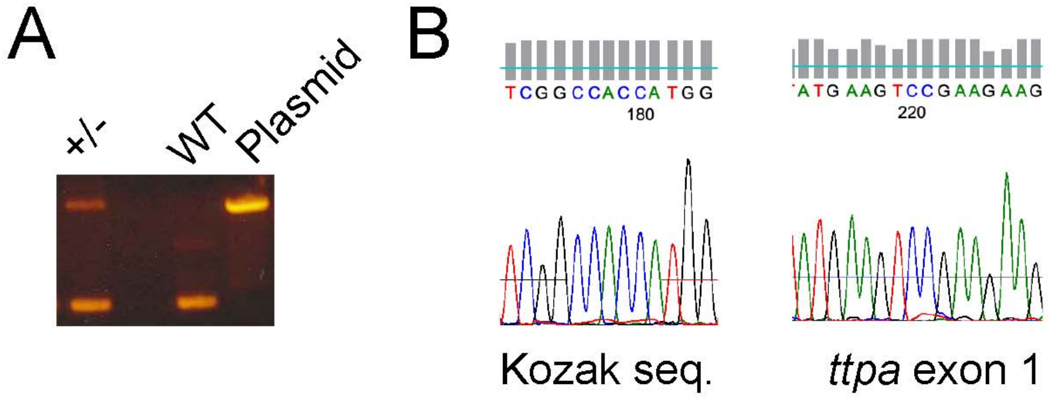

Supplementary Figure S1. Genotyping confirmation indicates successful genomic integration in heterozygote RedEfish. (A) PCR products generated from heterozygote (+/-) F3 RedEfish and Casper (WT) fin clips. Plasmid sequence used for positive control of mScarlet cassette. (B) Sanger sequencing confirmed no insertions or deletions 5' of the mScarlet coding sequence (Kozak seq.) and in the first exon of *ttpa*.

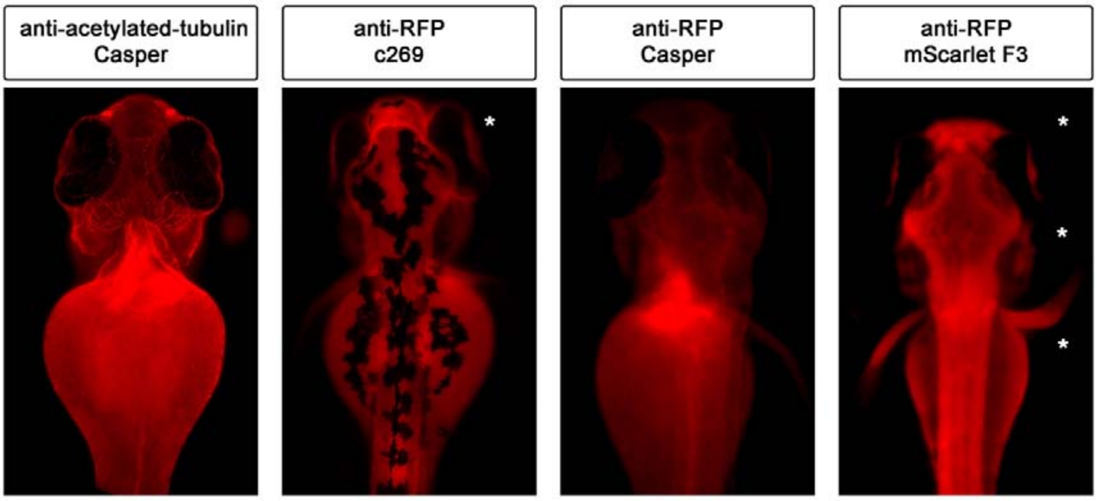

Supplementary Figure S2. RFP expression confirmation in 3 days post-fertilization (dpf) c269, Casper and RedEfish. Anti-acetylated tubulin was used as a control for the assay. c269 embryos express red fluorescent protein (RFP) in their mid- and hindbrain indicated with *. Casper embryos do not express RFP, however, autofluorescence and non-specific binding of the antibody is found just posterior of the yolk sac at 3 dpf. RedEfish (noted here as mScarlet F3) express RFP in the pectoral fins, mid- and hindbrain, down the trunk and partially in the yolk syncytium (*) at 3 dpf. Figure panels were generated with the BZ-x700 microscope, processed with BZ-X Analyzer Software with image adjustments equally applied across all images in Adobe Photoshop. All images are taken at 10x magnification at the same exposure protocol. This figure was created with Adobe Photoshop, v21.2.1.
